# Single-Cell RNA Sequencing Reveals Microevolution of the Stickleback Immune System

**DOI:** 10.1093/gbe/evad053

**Published:** 2023-04-11

**Authors:** Lauren E Fuess, Daniel I Bolnick

**Affiliations:** Department of Biology, Texas State University; Department of Ecology and Evolutionary Biology, University of Connecticut; Department of Ecology and Evolutionary Biology, University of Connecticut

**Keywords:** evolutionary immunology, host–parasite interactions, ecoimmunology

## Abstract

The risk and severity of pathogen infections in humans, livestock, or wild organisms depend on host immune function, which can vary between closely related host populations or even among individuals. This immune variation can entail between-population differences in immune gene coding sequences, copy number, or expression. In recent years, many studies have focused on population divergence in immunity using whole-tissue transcriptomics. But, whole-tissue transcriptomics cannot distinguish between evolved differences in gene regulation within cells, versus changes in cell composition within the focal tissue. Here, we leverage single-cell transcriptomic approaches to document signatures of microevolution of immune system structure in a natural system, the three-spined stickleback (*Gasterosteus aculeatus*). We sampled nine adult fish from three populations with variability in resistance to a cestode parasite, *Schistocephalus solidus*, to create the first comprehensive immune cell atlas for *G. aculeatus.* Eight broad immune cell types, corresponding to major vertebrate immune cells, were identified. We were also able to document significant variation in both abundance and expression profiles of the individual immune cell types among the three populations of fish. Furthermore, we demonstrate that identified cell type markers can be used to reinterpret traditional transcriptomic data: we reevaluate previously published whole-tissue transcriptome data from a quantitative genetic experimental infection study to gain better resolution relating infection outcomes to inferred cell type variation. Our combined study demonstrates the power of single-cell sequencing to not only document evolutionary phenomena (i.e., microevolution of immune cells) but also increase the power of traditional transcriptomic data sets.

**Significance**

Variation in immunological function is significant within and between species, yet the underlying mechanisms of this variation remain poorly understood, particularly at the cellular level. Here, we demonstrate significant variation in immune cell repertoires among closely related populations of the three-spined stickleback, *Gasterosteus aculeatus*. We also apply newly identified markers of immune cell types to reinterpret past immunological studies which used traditional transcriptomics. This reinterpretation suggests that differences in cell populations may be linked to differences in immune function. Our study is to our knowledge the first to document microevolution of immune cells, highlighting the power of single-cell approaches for studying immunity in nonmodel organisms.

## Introduction

Pathogenic infection is a major ecological interaction that drives physiological and immune response in hosts, natural selection (Cagliani and Sironi 2013; Gignoux-Wolfsohn et al. 2021), and population dynamics (Frick et al. 2010; Hochachka et al. 2021). Immense natural inter- and intraspecific variation exists in organismal response to pathogens (Lazzaro et al. 2004; Fuess et al. 2017; Grab et al. 2019), contributing significantly to disparate infection outcomes (Ellison et al. 2014; Fuess et al. 2017; Grab et al. 2019). While the consequences of variability in immunity are well documented, the underlying mechanisms which produce this variability are poorly understood. Historically, inter- and intraspecific variation in pathogenic response has been most often studied in the context of single components of the immune system (cells, genes, etc.) (Lazzaro et al. 2004; Schröder and Schumann 2005; Shinkai et al. 2012; Schenekar and Weiss 2017; Pérez-Espona et al. 2019). For example, the MHC II allele repertoire is significantly correlated to amphibian susceptibility to fungal pathogens; MHC heterozygosity across and within populations significantly affects pathogen resistance (Savage and Zamudio 2011). However, recent studies have suggested that intraspecific immune variation extends beyond single components to the broad cellular structure of immune systems. Studies have documented lineage-specific loss of immune cell types, as well as evolution of novel cell types in some species (Hilton et al. 2019; Guslund et al. 2020). This suggests that broad-scale variation in immune cell function and/or relative abundance might contribute to variation in immune responses. Still, the majority of data to this effect come at the among-species level or even larger macroevolutionary scales; less is known about the extent to which immune cell identity and function evolve at short time scales within species. Understanding the extent of immune cell microevolution is a necessary first step in deciphering how microevolution of immune cell types may contribute to divergence in immune response and pathogen resistance at a population level.

The immunological mechanisms underlying variable pathogen response and resistance remain particularity enigmatic in natural, nonmodel systems where most conclusions regarding differentiation in immunity are drawn from transcriptomic data generated from whole tissue samples (Dheilly et al. 2014; Sudhagar et al. 2018). While a powerful tool, traditional RNA sequencing (RNA-seq) studies condense any cell type heterogeneity within a sample to one data point. Thus, it is difficult to distinguish whether changes observed reflect regulatory changes in gene expression, versus shifting cell type abundance within the broader tissue. This problem is especially acute for immunological studies given the mobility of, and rapid mitotic diversification of, certain cell types and when considering nonmodel species for which genetic markers of prominent cell types are lacking.

Here, we leverage recent advances in single-cell RNA sequencing (scRNA-seq) technologies to test whether significant variation in immune cell abundance and/or function exists at the population level, potentially contributing to differentiation of immune responses. We focus our efforts on the emerging natural immunological model system, the three-spined stickleback (*Gasterosteus aculeatus*). This small fish is a tractable natural system for considering questions related to evolutionary and ecological immunology, largely due to their unique natural history. During the Pleistocene deglaciation, ancestrally anadromous populations of stickleback became trapped in newly created freshwater lakes (McKinnon and Rundle 2002). Thousands of independent lake populations have since been evolving in response to novel biotic and abiotic stimuli associated with freshwater environments for thousands of generations. This transition to freshwater exposed stickleback to many new parasites, including freshwater-exclusive, cestode parasite, *Schistocephalus solidus* (Simmonds and Barber 2016). Populations have subsequently evolved different immune traits to resist or tolerate this parasite (Weber et al. 2017a). Immense variation exists between independent lake populations in susceptibility to *S. solidus* (Weber et al. 2017b, 2022). Consequently, the *G. aculeatus*–*S. solidus* system provides a great opportunity for addressing diverse questions related to evolutionary and ecological immunity. Despite this opportunity, the understanding of the broader structure of the stickleback immune system (i.e., immune cell types and functions) is limited. We conducted scRNA-seq analysis to advance our understanding of immune cell repertoires and function in this important natural model system. Additionally, we leveraged the unique natural history of this species to assess questions regarding the response of immune systems to selective pressure (i.e., a novel parasite). By comparing immune cell repertoires among ecologically divergent but closely related populations of fish, we are able to demonstrate that selection can create rapid evolutionary change in not only relative immune cell abundance but also function (i.e., gene expression) of these immune cell types. These findings add further evidence that variation in broad immune system structure contributes to functional diversity of immunity and divergence in immune responses on a microevolutionary scale.

## Results and Discussion

### The Stickleback Head Kidney is Comprised of Eight Cell Types

To create a description of the immune cell repertoire of the three-spined stickleback, *G. aculeatus*, we conducted scRNA-seq and associated analysis of nine laboratory-raised adult fish. Individuals were lab-raised descendants bred from wild-caught ancestors from three different populations on Vancouver Island with variable resistance to *S. solidus* (3 fish per population). These populations include one anadromous population from Sayward Estuary, which are highly susceptible to *S. solidus* which they rarely encounter in nature (Weber et al. 2017a). In Gosling Lake, fish are frequently infected and tolerate rapid tapeworm growth (Weber et al. 2022). In the nearby Roberts Lake, the parasite is extremely rare, apparently because the fish are able to mount a strong fibrosis immune response that suppresses tapeworm growth and can even lead to parasite elimination. The three populations have been diverging in isolation for ∼12,000 years (since Pleistocene deglaciation) and exhibit weak but significant differences in allele frequencies throughout the genome, most strongly at loci under divergent selection (Weber et al. 2022). Prior work on these populations, including experimental infection of pure genotypes, F1 hybrids, and a recombinant F2 mapping population (backcrosses and intercrosses) confirmed that there are heritable differences in resistance to *S. solidus* (Weber et al. 2022). Flow cytometry and whole-tissue transcriptomics of the pronephros (a.k.a. “head kidney,” an important hematopoietic organ in fish) (Kum and Sekkin 2011), identified genetic associations between infection outcomes and cell type composition (lymphocyte/leukocyte ratios, coarsely defined) and gene expression (Fuess et al. 2021b; Weber et al. 2022). Here, we revisit this result by first generating single-cell RNA-seq data from the three focal populations. Importantly, the fish sampled for the scRNA-seq were not infected with this cestode parasite but instead represent constitutive population-level variability. Resulting libraries ranged in size from 8,119 to 19,578 cells with mean reads per cell ranging from 15,580 to 55,204 and median genes per cell ranging from 307 to 707. Mapping rates of genes to the newest version of the stickleback genome (Peichel et al. 2017, 2020; Nath et al. 2021) with improved annotations ranging from 51% to 68%, with most samples mapping at >55%. The use of improved annotations reduces but does not eliminate the impacts of 3′-UTR bias on our data analysis, an important consideration for future studies (Healey et al. 2022). Following filtering (see **Methods** for details), our final data set consisting of samples ranges between 1,780 and 9,160 cells per library. First, we describe these scRNA-seq results which provide the first immune cell atlas for *G. aculeatus*, then we use this new resource to re-examine the prior experimental infection data.

**Fig. 1.**
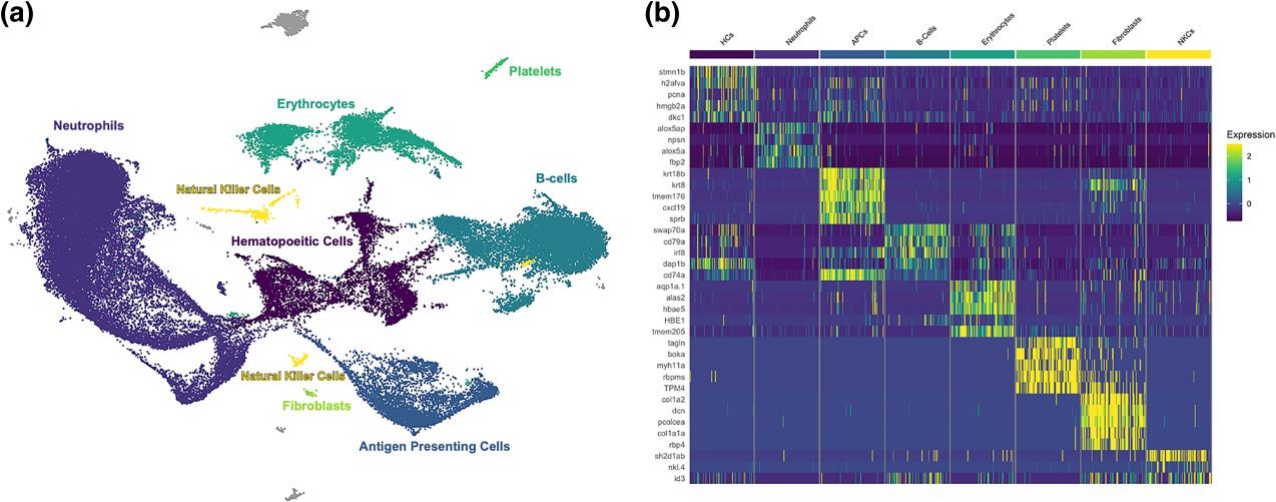
Summary of major cell types including identifying marker genes. *a*) UMAP projection of head kidney cells generated from combining all nine samples. Each point represents a single cell. Cells are color coded by their cluster and annotated cell type. Cells are shown grouped into broad cell type clusters based on distinguishing genes. For original cluster assignments, see supplementary fig. S1–2 and table S1, Supplementary Material online. *b*) Heatmap of the top five annotated distinguishing genes per cluster. Scaled expression, generated using the Seurat R package, is displayed for each gene. Cells are grouped by type, and genes are listed in order of significance. Only three annotated genes were significant for the NKC cluster.

A first pass analysis of the resulting data revealed 24 unique clusters of cells. However, further analysis of these clusters and their marker genes revealed that many of these original 24 clusters likely corresponded to subtypes (or different activation states) of major vertebrate immune cell types. Based on key marker genes (supplementary table S1, Supplementary Material online) each of these original clusters was assigned a putative cell type. Clusters assigned to the same cell type were then condensed, resulting in eight new clusters (fig. 1; supplementary figs. S1–S2, table S1, File 1, Supplementary Material online). These eight new clusters were representative of most major immune cell types (Carmona and Gfeller 2018): hematopoietic cells (HCs), neutrophils, antigen-presenting cells (APCs), B-cells, erythrocytes (RBCs), platelets, fibroblasts, and natural killer cells (NKCs) (supplementary figs. S3–S10, File 2, Supplementary Material online). Most of the original 24 unique clusters were easily grouped into one of these eight major groups based on comparison to existing data regarding vertebrate and teleost immune cell expression. For example, highly abundant neutrophils bear strong similarity to previously described teleost neutrophils, including high expression of zebrafish neutrophil marker *nephrosin* (*npsn*) (Di et al. 2017) (supplementary figure S4, Supplementary Material online). APCs were marked by high expression of group-specific genes involved in the presentation of antigens via the MHC II system (Al-Daccak et al. 2004; Roche and Furuta 2015) and low expression of B-cell marker genes such as *cd79a* (Chu and Arber 2001) (supplementary figure S5, Supplementary Material online). Also present in low abundance were a number of important immune cell types: platelets, fibroblasts, and NKCs; all of which were easily identifiable based on high expression of characteristic genes (supplementary figs. S8–S10, Supplementary Material online). The cluster was comprised of two distinct original clusters which were both characterized by high expression of NKC markers. Unfortunately, these two subgroups were not easily distinguished due to low representation. However, there were subtle differences in gene expression between the two groups which could be determined: one of these subgroups displayed constitutive expression of the human innate lymphoid cell (ILC) marker gene, *rorc* (Hoorweg et al. 2012), as well as high expression of *runx3*, which modulates development of ILCs (Ebihara et al. 2015), providing some support that this subgroup was comprised of putative fish ILCs. Conspicuously absent were putative T-cells. This can likely be explained due to the nature of the pronephros, which is believed to operate similarly to mammalian bone marrow (Tomonaga et al. 1973; Hitzfeld et al. 2005; Kum and Sekki 2011). Consequently, T-cells are likely only transiently found in this organ, perhaps primarily early in life. Alternatively, T-cells may have been less robust to the cell isolation procedure.

### Stickleback RBCs Express a Variety of Immune Genes

**Fig. 2.**
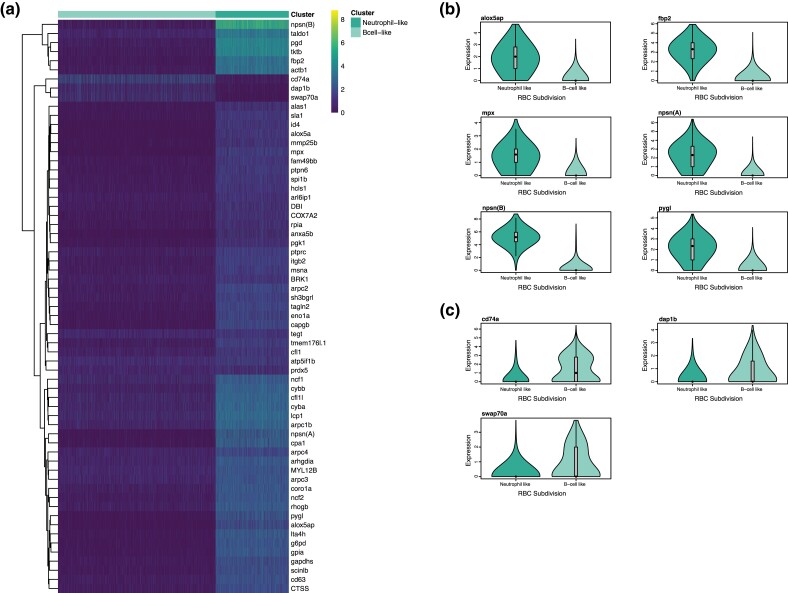
Differential expression of immune genes among the two identified RBC subgroups (neutrophil like and B-cell like). *a*) Heatmap of log-normalized expression of annotated B-cell and neutrophil marker genes which were significantly differentially expressed between the two RBC subgroups (mitochondrial and ribosomal genes excluded). Heatmap generated using the pheatmap package in R. *b*) Violin plot of log-normalized expression of significantly differentially expressed neutrophil marker genes among the two subgroups of cells. *c*) Violin plot of log-normalized expression of significantly differentially expressed B-cell marker genes among the two subgroups of cells.

In teleosts, unlike mammals, RBCs are nucleated and genetically active (Witeska 2013). A large, heterogenous group of cells with high expression of hemoglobin-associated genes was identified as putative RBCs. Interestingly, these cells also had high expression of a number of immune genes characteristic of both neutrophils and B-cells (fig. 2). Previous findings have indicated that teleost RBCs have diverse roles in the regulation of host immunity (Pereiro et al. 2017; Shen et al. 2018). For example, it is well documented that teleost RBCs contribute to antiviral immunity (Nombela et al. 2017; Pereiro et al. 2017; Puente-Marin et al. 2019). Preliminary evidence suggests they also can phagocytose and kill bacterial pathogens (Qin, et al. 2019) and even yeast (Passantino et al. 2002). However, our results suggest further refinement of these functions. Clustering analysis shows two distinct subgroups of RBCs, dividing based on similarity to either myeloid- (neutrophil) or lymphoid- (B-cells) type cells (fig. 2). Thus, while previous studies have both characterized myeloid-type functions (Pereiro et al. 2017; Puente-Marin et al. 2019; Qin et al. 2019) and document interactions with lymphoid cells (Jeong et al. 2016), this is the first evidence for diversification of teleost RBCs into distinct subgroups, each serving a particular immunological role. Further study is needed to improve the understanding of the distinct roles of these two subtypes and their broad roles in fish immunity.

### Two Groups of B-Cells Are Identifiable: Resting and Plasma B-Cells

A large group of cells uniquely expressing *cd79a*, *swap70a*, and a number of putative immunoglobulin genes was identified as putative B-cells (fig. 1). This group was comprised of three subclusters (original clusters 11, 12, and 13; supplementary fig. S2, Supplementary Material online), two of which (cluster 12 and cluster 13) were readily distinguished by expression patterns (supplementary fig. S11, Supplementary Material online). The smaller of the two subclusters (cluster 13) had considerably higher expression of immunoglobulin genes as well as *X-box binding protein 1* (*xbp1*) and associated proteins, key markers of plasma cells in mammals (Shaffer et al. 2004). Thus, we concluded that these two groups likely comprised of resting B-cells (cluster 12) and activated/plasma B-cells (cluster 13), respectively. Previous work has documented the diversification of fish B-cells into antibody-secreting cells upon immune stimulation (Jenberie et al. 2018). Furthermore, studies have indicated that antibody-secreting cells (including plasma cells and plasmablasts) constitute a stable subpopulation of cells in the head kidney of other fish species. Interestingly though, low levels of resting B-cells in the head kidney have been documented in salmonids of stock origin, which is contrary to our results (Ma et al. 2013). High levels of resting B-cells are characteristic of tissues involved in inducible responses to immune challenge, typically the blood and spleen in teleost fish (Ma et al. 2013). However, it is possible that some fish lineages may have evolved more plasticity in head kidney function as part of an inducible immune response. Further characterization of B-cell subpopulation in other tissue types from *G. aculeatus* will provide insight regarding the lineage-specific roles of various lymphoid tissues in immunity.

### Isolated Populations of Stickleback Vary Significantly in Cell Type Abundance

**Fig. 3.**
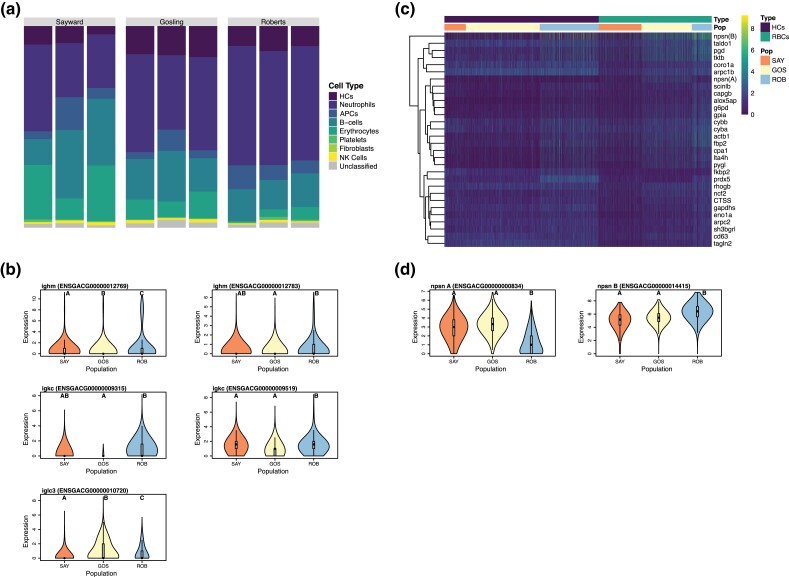
Summary of variation in immune cell subtype abundance and expression across three sampled populations of *G. aculeatus*. *a*) Bar graph representing relative abundance of each of the eight major cell types within each of the three sampled populations. *b*) Comparison across populations (SAY, Sayward; GOS, Gosling; ROB, Roberts Lake fish) of expression of B-cell–specific expression of putative immunoglobulin genes. All five genes were significantly differentially expressed between two or more populations. Letters indicate statistically significant groups. *c*) Heatmap of expression of neutrophil marker genes in both the HCs and erythrocytes. Columns are clustered by cell type and then population. Genes are clustered by similarity of expression profile using base algorithms from the pheatmap package in R. *d*) Comparison of neutrophil-specific expression of the two copies of nephrosin across the three sampled populations. Letters indicate statistically significant groups.

The nine fish sampled for our scRNA-seq analysis were representative of three isolated and genetically divergent populations. Laboratory experimental infection studies have confirmed that these three populations, Roberts Lake, Gosling Lake, and Sayward (anadromous) differ considerably in their immune responses to a common freshwater parasite, *S. solidus* (Weber et al. 2017b, 2022). The marine population is evolutionarily naïve to the parasite, which does not survive brackish water, and consequently is readily infected and permits rapid cestode growth. Both Gosling and Roberts Lakes are more resistant to laboratory infection than their marine ancestors, but the most resistant Roberts Lake population significantly suppresses cestode growth and is more likely to encapsulate and kill the cestode in a fibrotic granuloma (Weber et al. 2017b, 2022). Here, we tested whether the three populations exhibit differences in immune cell relative abundance, or differences in within-cell-type expression. We found significant between-population variation in abundance in every cell type except fibroblasts (supplementary table S2, Supplementary Material online; fig. 3). Roberts Lake fish (ROB), which are most resistant to the parasite, had considerably more neutrophils and platelets, but significantly less NKCs, RBCs, and B-cells than the other two populations. Sayward fish (parasite naïve) had the highest abundance of APCs, B-cells, and RBCs. It is important to note that sampled fish did vary in age, which may have had some effect of observed variation in immune cell repertoires. Furthermore, the context of this study does not allow for any firm conclusions linking these single-cell differences to functional effects on, or adaptation to, specific parasite species. While it is likely the differences reflect adaptive divergence due to natural selection (given the populations’ recent divergence), the three populations differ with respect to multiple environmental factors and multiple parasite species (Bolnick et al. 2020) so attributing evolution to a specific parasite requires future confirmation. Such confirmation might entail experimental coevolution of the host and parasite, or comparative methods spanning numerous populations to establish a reliable correlation between a given gene change and a particular parasite. Still, several of the observed differences in immune cell repertoires across populations could have significant implications for host defense. For example, ROB had higher abundance of neutrophils and platelets, both of which play important roles in parasite defenses. Neutrophils and other granulocyte cells such as eosinophils are important components of the initial innate immune response to helminths and other parasites (Chen et al. 2014; El-Naccache et al. 2020). Platelets, specifically thrombocyte-derived compounds, are important mediators of fibrotic responses (Antoniades et al. 1990; Abdollahi et al. 2005), and fibrosis is a major part of Roberts Lake sticklebacks’ response to *S. solidus* infection (Weber et al. 2022). Consequently, enhanced abundance of both neutrophils and platelets in ROB may allow for quick induction of resistance phenotypes (i.e., fibrosis) (Hund et al. 2022) and other immune responses which result in the efficient elimination of the parasite. It should be noted that the lack of variation in fibroblast abundance among populations is not unexpected; the fish used here are uninfected and so do not differ in fibrosis levels. Also, while platelets normally originate in hematopoietic tissues, like the head kidneys (Chang et al. 2007), fibroblasts are usually stimulated at sites of damage (Wynn 2008), which is in the peritoneal (body) cavity for the *S. solidus* parasite. Combined, the differences in relative abundance of immune cell types observed among our three populations of fish are likely to be mechanistically linked to observed variation in parasite resistance, an inference we revisit below by reanalyzing prior experimental data.

### Expression of Each Cell Type Varies among Populations

In contrast to the significant variation in relative abundance of immune cell types between the three sampled populations, we found modest signatures of among-population variation in expression profiles within cell types (supplementary File 3, Supplementary Material online). Most notable was variation in expression of immunoglobulin-like genes in B-cells (fig. 3). Despite having significantly fewer total B-cells (consistent with their lower proportion of lymphocytes in prior flow cytometry data) (Weber et al. 2017a), ROB had B-cells which exhibited higher average expression of immunoglobulin-type genes per cell. This may be a compensatory method as B-cell production of immunoglobulin is an essential component of response to helminth infection (Ma et al. 2013). Higher expression of immunoglobulin genes by Roberts Lake B-cells is likely the result of a significantly higher relative abundance of putative plasma B-cells in ROB (compared with resting B-cells). ROB, when compared with Gosling Lake and Sayward fish, had higher proportions of plasma cells. This was true when considering both the ratio of plasma cells to all head kidney cells, and plasma cells to B-cells specifically (*χ*^2^ test; *P*adj < 0.001). Again, this difference may be connected to variability in parasite resilience. Helminth-protective T_H_2-type immune responses induce expansion of plasma cells producing IgE (Anthony et al. 2007). Thus, a higher constitutive abundance of plasma-type B-cells in ROB may contribute to enhanced resistance to *S. solidus* parasites.

Finally, patterns of expression of neutrophil-associated markers also varied significantly across populations. Both HCs and RBCs in Roberts Lake had significantly higher expression of neutrophil marker genes (fig. 3). This is likely the result of enhanced overall investment in neutrophil-like cells in ROB, which could support a quick initial response to invading parasites (Chen et al. 2014; El-Naccache et al. 2020). Perhaps most interestingly, we observed population-specific, preferential expression of what is presumably duplicated copies of the important zebrafish neutrophil marker gene, *npsn*. We identified two highly similar genes annotated as *npsn*, both of which were significant markers of neutrophils. However, one gene was preferentially expressed by ROB, while the other was expressed higher in Gosling and Sayward neutrophils (fig. 3). Sequence comparison of these two gene copies revealed that while highly similar to zebrafish *npsn*, there are several species-specific and copy-specific amino acid substitutions in the sequences, suggesting potential neofunctionalization (supplementary fig. S12, Supplementary Material online). Neofunctionalization of one copy of this gene could be the result of any number of environmental differences between the populations but is of particular interest here due to the apparent speed at which shifts in preferential expression of these two isoforms have evolved.

### Insights from scRNA-seq Analyses Improve Interpretation of Past Traditional RNA-seq Studies

**Fig. 4.**
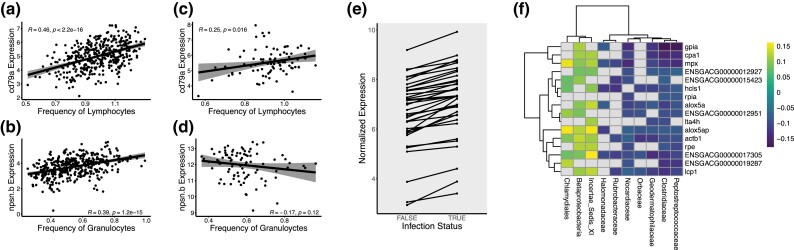
Evaluation of applicability of identified markers to past traditional RNA-seq data sets *a*–*d*) Pearson correlations between expression of identified lymphocyte (cd79*a*) or granulocyte markers (npsn.b) and normalized lymphocyte or granulocyte frequency (detected by flow cytometry) in our two previous transcriptomic study sets (*a, b*) (Fuess et al. 2021b) (*c, d*) (Lohman et al. 2017). For all correlation plots, the regression line is shown and shading indicates 95% confidence intervals. *e*) Patterns of differences in gene expression of identified APC in uninfected versus infected fish: all data shown correspond to genes which were significantly differentially expressed in a previous traditional RNA-seq study (Fuess et al. 2021b). *f*) Heatmap of significant correlations (tau) between gene expression of identified neutrophil markers and abundance of specific microbial taxa. Nonsignificant correlations are displayed in grey. Data taken from a previous correlative analysis of traditional RNA-seq data (Fuess et al. 2021a).

The scRNA-seq data allowed us to confidently identify a suite of genes which are markers of each of these putative eight cell types (supplementary File 2, Supplementary Material online). Using these new candidate marker genes, we can reevaluate findings of past RNA-seq studies to understand the relative contributions of changes in gene expression versus changes in cell abundance. Specifically, we leveraged these markers to reinterpret results from two previous studies for which we had both traditional RNA-seq expression data and flow cytometry data coarsely estimating granulocyte to lymphocyte relative abundance using forward and side-scatter gating (Lohman et al. 2017; Fuess et al. 2021b). The first, and larger, of the two studies investigated variation in constitutive and induced immune response to experimental parasite infections in laboratory-reared F2 fish (Fuess et al. 2021b). Within this data set, granulocyte and lymphocyte frequencies are, respectively, correlated to expression of both putative granulocyte markers (*npsn* B, transcript 1; Pearson correlation, *P* < 0.001, *r* = 0.3904) and lymphocyte markers (*cd79a*; Pearson correlation, *P* < 0.001, *r* = 0.4569). The second, smaller, study conducted a similar experimental parasite infection of laboratory-reared F1 fish (Lohman et al. 2017). Within this study, these correlations are less significant for lymphocytes (Pearson correlation, *P* = 0.016, *r* = 0.25), and both nonsignificant and trending in the opposite direction for granulocytes (Pearson correlation, *P* = −0.17, *r* = 0.12; fig. 4). These inconsistencies are likely due to the nature of our correlative data. Flow cytometry grouped cells into two large bins: granulocytes and lymphocytes. Thus, finding two markers that accurately correlate to these broad groups across experiments is difficult, particularly for diverse granulocytes. Examination of a broader group of potential markers revealed strong correlations between several additional markers and both lymphocyte granulocyte abundances for our larger data set, and strong associations between additional lymphocyte markers and lymphocyte abundance for our smaller data set (supplementary figs. S13–14, Supplementary Material online. These findings suggest that variation in expression of cell markers identified here may be reflective of changes in abundance of immune cell types. We believe that further validation using more comprehensive paired transcriptomic and flow cytometry data will demonstrate that this data provide a powerful new resource that will increase the interpretive power of traditional RNA-seq analyses, particularly when combined with developing methods for deconvolution of bulk-tissue data (Cobos et al. 2020; Jin and Liu 2021).

Assuming that changes in expression of these markers is at least in part due to changes in their respective cell type, we can now glean more insight regarding the cellular changes in response to infection of *G. aculeatus* by *S. solidus* by reexamining previous data sets. Consequently, we applied the markers generated here to reinterpret results from the two studies of response experimental parasite infection in laboratory-reared F1 and F2 fish (Lohman et al. 2017; Fuess et al. 2021b). In each case, we conducted *χ*^2^ tests to detect overrepresentation of cell markers (generally or specific cell type) among significantly differentially expressed genes. In the case of groups where significant overrepresentation was detected, we conducted a proportion test to detect statically significant skew in the directionality of differential expression. In the smaller study of response of laboratory-reared F1 fish, we observed few significant patterns of biological interest (Lohman et al. 2017); supplementary table S3, Supplementary Material online). However, in our larger data set (F2 fish), we noticed significant overrepresentation of APCs and B-cell marker genes among the genes differentially expressed as a result of infection or between populations, respectively (Fuess et al. 2021b) (supplementary table S3, Supplementary Material online). Importantly, this result is in F2 hybrid fish where recombination during two generations of breeding has randomized most between-population differences, and environmental effects are removed by rearing two generations in the lab. Thus, associations between our interpolated cell type results and infection outcomes represent evidence for genetic covariance between the cell type (expression) and resistance traits. Markers of APCs were not only significantly overrepresented but also exclusively increased in response to infection (fig. 4). Alternatively activated macrophages are known to play key roles in response to helminth infection, including mediating inflammatory responses (Kreider et al. 2007; Coakley and Harris 2020). B-cell markers were generally expressed at higher levels in susceptible back-crossed fish compared with resistant back-crosses, consistent with analysis of scRNA data presented here.

Finally, we also considered results from correlative analyses of associations between gene expression in F2 fish, and gut microbiome composition (Fuess et al. 2021a). Here, we observed significant overrepresentation of markers of neutrophil, B-cell, and fibroblast cells among lists of genes significantly correlated to abundance of specific microbial taxa in the gut (supplementary table S3, Supplementary Material online). Neutrophils demonstrated the most consistent patterns of association with microbial taxa abundance, with some microbial taxa demonstrating strongly significant positive or negative associations with many neutrophil markers (fig. 4). Neutrophils and gut microbiota are believed to be functionally linked, with gut microbiota regulating components of neutrophil activity and vice versa (Lajqi et al. 2020). Our findings suggest that specific microbiota have systemic effects on the proliferation of (or lack thereof) neutrophils in hematopoietic organs. In sum, the markers discovered here provide new power to interpret traditional RNA-seq data and begin to disentangle relative contributes of changes in gene expression versus changes in cell type abundance. These results point to the value of small-sample scRNA-seq in guiding reinterpretation of new or existing large-sample bulk-tissue transcriptomic data and hence the potential future value in emerging methods for bulk-RNA-seq deconvolution (Cobos et al. 2020; Jin and Liu 2021).

## Conclusions

Here, we present a robust analysis of population-specific variation in immune system structure (relative cell type abundance and function) and the potential connection of this variation with observed variation in parasite resistance. Using single-cell RNA-seq analyses, we demonstrate that independent populations, with known differences in parasite resistance, vary significantly in both abundance and expression patterns of immune cell types. Our reanalysis of prior bulk tissue data then allows us to infer cell-type correlations with the results of experimental infections (cestode infection success and growth). This is, to our knowledge, the first evidence that rapid evolution of immune cell repertoires among populations both occurs and potentially contributes to variation in immune response and infection outcome. Our results add to the growing body of evidence that suggests that the immune system may be much more malleable than once thought. Furthermore, these findings provide compelling rationale for further studies investigating adaptability of immune system structure within and between species, focusing on the evolutionary causes of such adaptability. Also notably, our findings present the first description of prominent immune cell types in an important ecological and evolutionary model species. This provides new cell marker resources that can be used to streamline further immunological studies and provide new insight into traditional RNA-seq studies. In sum, our work not only adds strong evidence suggesting that microevolution of immune cell repertoires contributes to variation in immune response but also provides a robust new tool for researchers utilizing the stickleback system as a model of evolutionary and ecological immunology.

## Methods

### Sample Collection & Processing

Single-cell libraries were generated from head kidneys of laboratory-reared F1 stickleback from three populations on Vancouver Island in British Columbia (Sayward Estuary, Roberts Lake, and Gosling Lake). Reproductively mature fish were collected at each location using minnow traps. Gravid females were stripped of their eggs, which were then fertilized using sperm obtained from macerated testes of males from the same lake. Fish were collected with permission from the Ministry of Forests, Lands, and Natural Resource Operations of British Columbia (Scientific Fish Collection permit NA12-77018 and NA12-84188). The resulting eggs (F1 generation) were shipped back to Austin, Texas, hatched, and reared to maturity in controlled laboratory conditions. At ∼2–3 years of age, fish were transferred to aquarium facilities at the University of Connecticut. At the time of sampling, fish ranged from 3 (Sayward and Gosling) to 4 (Roberts) years of age. Sampled fish were a random selection of F1-generation fish of unknown relatedness. All sampled fish were male.

We generated single-cell suspensions from the pronephros (head kidney) of three fish from each population (Sayward, Roberts, and Gosling). Fish were humanely euthanized one at a time, and their head kidneys were immediately extracted. Dissected head kidneys were placed in 2 mL of R-90 media (90% RPMI 1640 with L-glutamine, without Phenol red; Gibco) in a sterile 24-well plate on ice. Tissue was then physically dissociated using a sterile pipette tip. The resulting slurry was then strained through a 40 *μ*m nylon filter. An additional 2 mL R-90 was added to the resulting suspension. Cells were then spun at 440 *g* for 10 min at 4 °C. The supernatant was removed, and cells were resuspended in 2 mL R-90. Cells were spun one more time, and the resulting supernatant was replaced with 1 mL R-90. Cell suspensions were then transported on ice to the Jackson Lab facility in Hartford, Connecticut, where samples were prepared for sequencing and sequenced within 6 h of initial sample collection.

### Single-Cell Library Preparation and Sequencing

Cells were washed and suspended in PBS containing 0.04% BSA and immediately processed as follows. Cell viability was assessed on a Countess II automated cell counter (ThermoFisher), and an estimated 12,000 cells were loaded onto one lane of a 10× Genomics Chromium Controller. Single-cell capture, barcoding, and single-indexed library preparation were performed using the 10× Genomics 3′ Gene Expression platform version 3 chemistry and according to the manufacturer's protocol (#CG00052) (Zheng et al. 2017). cDNA and libraries were checked for quality on Agilent 4200 Tapestation, quantified by KAPA qPCR, and sequenced on an Illumina sequencer which targeted 6,000 barcoded cells with an average sequencing depth of 50,000 read pairs per cell. Three initial libraries (one per population) were sequenced on individual lanes of a HiSeq 4000 flow cell; all other libraries were sequenced on a NovaSeq 6000 S2 flow cell, each pooled at 16.67% of the flow cell lane.

Illumina base call files for all libraries were converted to FASTQs using bcl2fastq v2.20.0.422 (Illumina), and FASTQ files were aligned to reference genome constructed from the v5 G. aculeatus assembly and annotation files available at https://stickleback.genetics.uga.edu/(Nath, et al. 2021). Briefly, annotations from Ensembl (release 95) were combined with repeat, Y chromosome, and revised annotations from Nath et al. using AGAT (0.4.0) (Dainat et al. 2021), and a STAR-compatible reference genome was generated by Cell Ranger (v3.1.0, 10× Genomics) using these annotations and the v5 assembly from Nath et al. The Cell Ranger count (v3.1.0) pipeline was used to construct the cell-by-gene counts matrix for each library, subsequently analyzed using Scanpy 1.3.7 (Wolf et al. 2018) and the Loupe Cell Browser (10× Genomics).

Each counts matrix was individually subjected to quality control filtering, such that cells with more than 35,000 UMIs, fewer than 400 genes, more than 30% mtRNA content, and more than 1,000 hemoglobin transcripts were discarded from downstream analysis. The nine filtered counts matrices were concatenated, normalized by per-cell library size, and log transformed. The expression profiles of each cell at the 4,000 most highly variable genes (as measured by dispersion) (Satija et al. 2015; Zheng et al. 2017) were used for principal component (PC) analysis and subsequently batch corrected using Harmony (Korsunsky et al. 2019). The batch-corrected PCs were utilized for neighborhood graph generation (using 25 nearest neighbors) and dimensionality reduction with UMAP (McInnes et al. 2020). Clustering was performed on this neighborhood graph using the Leiden community detection algorithm (Traag et al. 2019). Subclustering was performed on a per-cluster ad hoc basis to separate visually distinct subpopulations of cells. This UMAP embedding and clustering metadata were then imported into the Loupe Cell Browser (generated using Cell Ranger aggr [v3.1.0]) for interactive analysis.

### Cluster Identification

Once data (UMAP embedding and clustering metadata) were loaded into the Loupe Cell Browser, we then generated lists of marker genes for each of the identified clusters using the “Globally Distinguishing” feature. Marker genes were classified as those genes upregulated in each cluster (compared with all other cells) with an adjusted *P* < 0.10. Next, we assigned tentative identities to each of these initial clusters by comparison of marker genes to available literature regarding markers of immune cells in teleost fish and other vertebrates. During this initial identification process, we identified multiple groups of cells with homology to the same major immune cell type (e.g., three clusters demonstrated patterns of expression indicative of neutrophils). Consequently, we condensed the initial 24 identified clusters into eight major groups based on homology to known vertebrate immune cell types for downstream analyses. Supplementary table S1, Supplementary Material online, lists all original clusters, their top statistical marker genes, the genes used to assign the cluster to a major immune cell type, and the final immune cell type assignment. After this process, we also examined differential expression between original clusters within these major groups using the “Locally Distinguishing” feature in the Loupe Cell Browser. Cluster identification and subcluster distinctions were confirmed by visual analysis of expression of major immune cell type markers in the Loupe Cell Browser. Violin plots and heatmaps displaying patterns of expression across major group and subclusters within groups were generated in R using read count matrices and cluster identity information (exported from the Loupe Cell Browser). Relevant code can be found at https://github.com/lfuess/scRNAseq.

### Comparative Analyses Across Populations

When comparing across populations, we assessed two hypotheses: 1) relative abundance of immune cell types is variable across populations and 2) expression patterns within each identified immune cell type are variable across populations. First, to identify differences in relative abundance of each of our eight major immune cell types, we performed independent, binomial general linear models (GLM) for each cell type. The binomial GLM uses the numbers of observed instances of a given cell type, out of a known number of observed cells per fish, to calculate the proportion of that cell type (with appropriate binomial error) and test whether this proportion varies between factor groups (e.g., populations). Tukey's post hoc tests were used for pair-wise comparisons if significant differences were identified between populations (the code can be found at https://github.com/lfuess/scRNAseq). Second, to identify differences in gene expression patterns within each of our identified immune cell types, we again used the “Locally Distinguishing” feature in the Loupe Cell Browser. Cells within each major group were subdivided by population, and then, all possible pairwise comparisons of gene expression were conducted. When conducting pairwise comparisons, the Loupe Browser specifically normalizes for differences in cell abundance between groups using size factors to ensure that differences in average cell expression and abundance are not conflated. Genes with adjusted *P* < 0.10 were identified as significantly differentially expressed. Relevant violin plots and heatmaps were generated in R using read count matrices and cluster identity information (exported from the Loupe Cell Browser). The relevant code can be found at https://github.com/lfuess/scRNAseq.

### Sequence Alignment

In order to examine sequence divergence in the two identified copies of neutrophil marker gene, *npsn*, we conducted a multiple sequence alignment of both *npsn* transcripts from stickleback and the zebrafish *npsn* transcript sequence using the R package msa (Bodenhofer et al. 2015).

### Comparison to Past Analyses

We leveraged past transcriptomic analysis of the stickleback head kidney to assess whether whole tissue-measured expression of putative markers identified here could be used as a reliable metric of relative cell type abundance. We specifically analyzed two past transcriptomic data sets: 1) an analysis of laboratory-reared F1 fish from Roberts and Gosling Lakes experimentally exposed to parasites (Lohman et al. 2017) and 2) an analysis of laboratory-reared F2 and backcrossed fish, the offspring of fish from experiment 1, experimentally exposed to parasites (Fuess et al. 2021b). For both of these data sets, we had access to transcriptomic data detailing whole tissue expression of our putative cell markers, and flow cytometry data coarsely estimating granulocyte to lymphocyte relative abundance using forward and side-scatter gating. For each data set, we examined the correlation between normalized gene expression of putative markers and square root transformed frequency data for granulocytes or lymphocytes as appropriate.

Once we established that whole-tissue expression of putative cell markers was at least partially indicative of relative abundance of immune cell types, we then leveraged our newly identified cell markers to reinterpret three past transcriptomic studies of stickleback immunity: the two previously mentioned transcriptomic studies of F1 & F2/backcross fish to immune challenge (Lohman et al. 2017; Fuess et al. 2021b) and an additional study examining correlations between head kidney gene expression and gut microbiome composition (Fuess et al. 2021a). Specifically, we used chi-squared tests to identify significant overrepresentation of markers of any given cell type within lists of genes significantly differentially expressed as a result of traits of interest, or genes significantly correlated to microbial diversity/taxa of interest. *χ*^2^ tests were used to test for overrepresentation of each immune cell type within each list of genes independently.

## Supplementary Material

evad053_Supplementary_Data

## Data Availability

Relevant data and code can be found at https://github.com/lfuess/scRNAseq.

## References

[evad053-B1] Abdollahi A , et al 2005. Inhibition of platelet-derived growth factor signaling attenuates pulmonary fibrosis. J Exp Med. 201:925–935. 15781583 10.1084/jem.20041393 PMC2213091

[evad053-B2] Al-Daccak R , Mooney N, Charron D. 2004. MHC Class II signaling in antigen-presenting cells. Curr Opin Immunol. 16:108–113. 14734118 10.1016/j.coi.2003.11.006

[evad053-B3] Anthony RM , Rutitzky LI, Urban JF Jr, Stadecker MJ, Gause WC 2007. Protective immune mechanisms in helminth infection. Nat Rev Immunol 7: 975–987. 18007680 10.1038/nri2199 PMC2258092

[evad053-B4] Antoniades HN , et al 1990. Platelet-derived growth factor in idiopathic pulmonary fibrosis. J Clin Invest. 86:1055–1064. 2170444 10.1172/JCI114808 PMC296832

[evad053-B5] Bodenhofer U , Bonatesta E, Horejs-Kainrath C, Hochreiter S. 2015. Msa: an R package for multiple sequence alignment. Bioinformatics 31:3997–3999. 26315911 10.1093/bioinformatics/btv494

[evad053-B6] Bolnick DI , Resetarits EJ, Ballare K, Stuart YE, Stutz WE. 2020. Scale-dependent effects of host patch traits on species composition in a stickleback parasite metacommunity. Ecology 101:e03181. 10.1002/ecy.3181 PMC7757261 32880940

[evad053-B7] Cagliani R , Sironi M. 2013. Pathogen-driven selection in the human genome. Int J Evol Biol. 2013:204240. 10.1155/2013/204240 PMC3603197 23533945

[evad053-B8] Carmona SJ , Gfeller D. 2018. Deciphering the evolution of vertebrate immune cell types with single-cell RNA-Seq. In: Pontarotti P, editor. Origin and evolution of biodiversity. Cham: Springer International Publishing. p. 95–111.ss

[evad053-B9] Chang Y , Bluteau D, Debili N, Vainchenker W. 2007. From hematopoietic stem cells to platelets. J Thromb Haemost. 5(Suppl 1):318–327. 17635743 10.1111/j.1538-7836.2007.02472.x

[evad053-B10] Chen F , et al 2014. Neutrophils prime a long-lived effector macrophage phenotype that mediates accelerated helminth expulsion. Nat Immunol. 15:938–946. 25173346 10.1038/ni.2984 PMC4479254

[evad053-B11] Chu PG , Arber DA. 2001. CD79: a review. Appl Immunohistochem Mol Morphol. 9:97–106. 11396639 10.1097/00129039-200106000-00001

[evad053-B12] Coakley G , Harris NL. 2020. Interactions between macrophages and helminths. Parasite Immunol. 42:e12717. 10.1111/pim.12717 32249432

[evad053-B13] Cobos FA , Alquicira-Hernandez J, Powell JE, Mestdagh P, De Preter K. 2020. Benchmarking of cell type deconvolution pipelines for transcriptomics data. Nat Commun. 11:5650. 33159064 10.1038/s41467-020-19015-1 PMC7648640

[evad053-B14] Dainat J , Herenu D. pascal-git, 2021. NBISweden/AGAT: AGAT-v0.8.0. Zendodo.

[evad053-B15] Dheilly NM , et al 2014. No more non-model species: the promise of next generation sequencing for comparative immunology. Dev Comp Immunol. 45:56–66. 24508980 10.1016/j.dci.2014.01.022 PMC4096995

[evad053-B16] Di Q , et al 2017. Zebrafish nephrosin helps host defence against Escherichia coli infection. Open Biol. 7:170040. 28835569 10.1098/rsob.170040 PMC5577445

[evad053-B17] Ebihara T , et al 2015. Runx3 specifies lineage commitment of innate lymphoid cells. Nat Immunol. 16:1124–1133. 26414766 10.1038/ni.3272 PMC4618046

[evad053-B18] El-Naccache DW , Chen F, Chen N, Gause WC. 2020. The NET effect of neutrophils during helminth infection. Cell Host Microbe. 27:165–168. 32053785 10.1016/j.chom.2020.01.013 PMC7196278

[evad053-B19] Ellison AR , et al 2014. More than skin deep: functional genomic basis for resistance to amphibian chytridiomycosis. Genome Biol Evol. 7:286–298. 25539724 10.1093/gbe/evu285 PMC4316636

[evad053-B20] Frick WF , et al 2010. An emerging disease causes regional population collapse of a common north American bat species. Science 329:679–682. 20689016 10.1126/science.1188594

[evad053-B21] Fuess LE , et al 2021a. Immune gene expression covaries with gut microbiome composition in stickleback. mBio 12:e00145-21. 33947750 10.1128/mBio.00145-21 PMC8262870

[evad053-B22] Fuess LE , et al 2021b. Between-population differences in constitutive and infection-induced gene expression in threespine stickleback. Mol Ecol 30:6791–6805. 34582586 10.1111/mec.16197 PMC8796319

[evad053-B23] Fuess LE , Pinzon CJ, Weil E, Grinshpon RD, Mydlarz LD. 2017. Life or death: disease-tolerant coral species activate autophagy following immune challenge. Proc Biol Sci. 284:20170771. 28592676 10.1098/rspb.2017.0771 PMC5474081

[evad053-B24] Gignoux-Wolfsohn SA , et al 2021. Genomic signatures of selection in bats surviving white-nose syndrome. Mol Ecol. 30:5643–5657. 33476441 10.1111/mec.15813

[evad053-B25] Grab KM , et al 2019. Host tolerance and resistance to parasitic nest flies differs between two wild bird species. Ecol Evol. 9:12144–12155. 31832149 10.1002/ece3.5682 PMC6854101

[evad053-B26] Guslund NC , et al 2020. Single-cell transcriptome profiling of immune cell repertoire of the Atlantic cod which naturally lacks the major histocompatibility class II system. Front Immunol. 11:559555. 10.3389/fimmu.2020.559555 PMC7588623 33154745

[evad053-B27] Healey HM , Bassham S, Cresko WA. 2022. Single-cell Iso-sequencing enables rapid genome annotation for scRNAseq analysis. Genetics 220:iyac017. 35143654 10.1093/genetics/iyac017 PMC8893252

[evad053-B28] Hilton HG , et al 2019. Single-cell transcriptomics of the naked mole-rat reveals unexpected features of mammalian immunity. PLoS Biol. 17:e3000528. 10.1371/journal.pbio.3000528 PMC6894886 31751331

[evad053-B29] Hitzfeld B , et al 2005. Fish immune system. In: Assenmacher M, editors. Encyclopedic reference of immunotoxicology. Berlin, Heidelberg: Springer Berlin Heidelberg. p. 242–245.

[evad053-B30] Hochachka WM , Dobson AP, Hawley DM, Dhondt AA. 2021. Host population dynamics in the face of an evolving pathogen. J Anim Ecol. 90:1480–1491. 33821505 10.1111/1365-2656.13469 PMC8227824

[evad053-B31] Hoorweg K , et al 2012. Functional differences between human NKp44(-) and NKp44(+) RORC+ innate lymphoid cells. Front Immunol. 3. 10.3389/fimmu.2012.00072 PMC3342004 22566953

[evad053-B32] Hund AK , et al 2022. Population-level variation in parasite resistance due to differences in immune initiation and rate of response. Evolution Letters 6:162–177. 35386836 10.1002/evl3.274 PMC8966477

[evad053-B33] Jenberie S , et al 2018. Profiling Atlantic salmon B cell populations: cpG-mediated TLR-ligation enhances IgM secretion and modulates immune gene expression. Sci Rep. 8:3565. 29476080 10.1038/s41598-018-21895-9 PMC5824956

[evad053-B34] Jeong J-M , An CM, Kim M-C, Park C-I. 2016. Cooperation of erythrocytes with leukocytes in immune response of a teleost Oplegnathus fasciatus. Genes Genomics. 38:931–938.

[evad053-B35] Jin H , Liu Z. 2021. A benchmark for RNA-seq deconvolution analysis under dynamic testing environments. Genome Biol. 22:102. 33845875 10.1186/s13059-021-02290-6 PMC8042713

[evad053-B36] Korsunsky I , et al 2019. Fast, sensitive and accurate integration of single-cell data with Harmony. Nat Methods. 16:1289–1296. 31740819 10.1038/s41592-019-0619-0 PMC6884693

[evad053-B37] Kreider T , Anthony RM, Urban JF Jr, Gause WC 2007. Alternatively activated macrophages in helminth infections. Curr Opin Immunol 19: 448–453. 17702561 10.1016/j.coi.2007.07.002 PMC2000338

[evad053-B38] Kum C , Sekki S. 2011. The immune system drugs in fish: immune function, immunoassay, drugs. In: Aral F and Do Z, editors. Recent advances in fish farms.London EnglandIntechOpen.

[evad053-B39] Lajqi T , Poschl J, Frommhold D, Hudalla H. 2020. The role of microbiota in neutrophil regulation and adaptation in newborns. Front Immunol. 11:568685. 10.3389/fimmu.2020.568685 PMC7550463 33133082

[evad053-B40] Lazzaro BP , Sceurman BK, Clark AG. 2004. Genetic basis of natural variation in D. melanogaster antibacterial immunity. Science 303:1873–1876. 15031506 10.1126/science.1092447

[evad053-B41] Lohman BK , Steinel NC, Weber JN, Bolnick DI. 2017. Gene expression contributes to the recent evolution of host resistance in a model host parasite system. Front Immunol. 8:1071. 28955327 10.3389/fimmu.2017.01071 PMC5600903

[evad053-B42] Ma C , Ye J, Kaattari SL. 2013. Differential compartmentalization of memory B cells versus plasma cells in salmonid fish. Eur J Immunol. 43:360–370. 23255215 10.1002/eji.201242570

[evad053-B43] McInnes L , Healy J, Melville J. 2020. UMAP: uniform manifold approximation and projection for dimension reduction. arXiv

[evad053-B44] McKinnon JS , Rundle HD. 2002. Speciation in nature: the threespine stickleback model systems. Trends Ecol Evol. 17:480–488.

[evad053-B45] Nath S , Shaw DE, White MA. 2021. Improved contiguity of the threespine stickleback genome using long-read sequencing. G3 Genes|Genomes|Genetics 11:jkab007. 33598708 10.1093/g3journal/jkab007 PMC8022941

[evad053-B46] Nombela I , et al 2017. Infectious pancreatic necrosis virus triggers antiviral immune response in rainbow trout red blood cells, despite not being infective. F1000Res 6:1968. 29333244 10.12688/f1000research.12994.1 PMC5747336

[evad053-B47] Passantino L , et al 2002. Fish immunology. I. Binding and engulfment of Candida albicans by erythrocytes of rainbow trout (Salmo gairdneri Richardson). Immunopharmacol Immunotoxicol. 24:665–678. 12510797 10.1081/iph-120016050

[evad053-B48] Peichel CL , et al 2020. Assembly of the threespine stickleback Y chromosome reveals convergent signatures of sex chromosome evolution. Genome Biol. 21:177. 32684159 10.1186/s13059-020-02097-x PMC7368989

[evad053-B49] Peichel CL , Sullivan ST, Liachko I, White MA. 2017. Improvement of the threespine stickleback genome using a hi-C-based proximity-guided assembly. J Hered. 108:693–700. 28821183 10.1093/jhered/esx058 PMC5892396

[evad053-B50] Pereiro P , et al 2017. Nucleated teleost erythrocytes play an Nk-Lysin- and autophagy-dependent role in antiviral immunity. Front Immunol. 8:1458. 29163526 10.3389/fimmu.2017.01458 PMC5673852

[evad053-B51] Pérez-Espona S , et al 2019. First assessment of MHC diversity in wild Scottish red deer populations. Eur J Wildlife Res. 65.

[evad053-B52] Puente-Marin S , et al 2019. Potential role of rainbow trout erythrocytes as mediators in the immune response induced by a DNA vaccine in fish. Vaccines (Basel). 7:60. 31277329 10.3390/vaccines7030060 PMC6789471

[evad053-B53] Qin Z , et al 2019. Antibacterial activity of erythrocyte from grass carp (Ctenopharyngodon idella) is associated with phagocytosis and reactive oxygen species generation. Fish Shellfish Immunol. 92:331–340. 31176765 10.1016/j.fsi.2019.06.008

[evad053-B54] Roche PA , Furuta K. 2015. The ins and outs of MHC class II-mediated antigen processing and presentation. Nat Rev Immunol. 15:203–216. 25720354 10.1038/nri3818 PMC6314495

[evad053-B55] Satija R , Farrell JA, Gennert D, Schier AF, Regev A. 2015. Spatial reconstruction of single-cell gene expression data. Nat Biotechnol. 33:495–502. 25867923 10.1038/nbt.3192 PMC4430369

[evad053-B56] Savage AE , Zamudio KR. 2011. MHC genotypes associate with resistance to a frog-killing fungus. Proc Natl Acad Sci U S A. 108:16705–16710. 21949385 10.1073/pnas.1106893108 PMC3189034

[evad053-B57] Schenekar T , Weiss S. 2017. Selection and genetic drift in captive versus wild populations: an assessment of neutral and adaptive (MHC-linked) genetic variation in wild and hatchery brown trout (Salmo trutta) populations. Conserv Genet. 18:1011–1022.

[evad053-B58] Schröder NWJ , Schumann RR. 2005. Single nucleotide polymorphisms of Toll-like receptors and susceptibility to infectious disease. Lancet Infect Dis. 5:156–164. 15766650 10.1016/S1473-3099(05)01308-3

[evad053-B59] Shaffer AL , et al 2004. XBP1, downstream of Blimp-1, expands the secretory apparatus and other organelles, and increases protein synthesis in plasma cell differentiation. Immunity 21:81–93. 15345222 10.1016/j.immuni.2004.06.010

[evad053-B60] Shen Y , Wang D, Zhao J, Chen X. 2018. Fish red blood cells express immune genes and responses. Aquaculture Fisheries. 3:14–21.

[evad053-B61] Shinkai H , et al 2012. Genetic variability in swine leukocyte antigen class II and Toll-like receptors affects immune responses to vaccination for bacterial infections in pigs. Comp Immunol Microbiol Infect Dis. 35:523–532. 22658914 10.1016/j.cimid.2012.05.003

[evad053-B62] Simmonds NE , Barber I. 2016. The effect of salinity on egg development and viability of Schistocephalus solidus (Cestoda: diphyllobothriidea). J Parasitol. 102:42–46. 26418088 10.1645/14-701

[evad053-B63] Sudhagar A , Kumar G, El-Matbouli M. 2018. Transcriptome analysis based on RNA-Seq in understanding pathogenic mechanisms of diseases and the immune system of fish: a comprehensive review. Int J Mol Sci. 19:245. 29342931 10.3390/ijms19010245 PMC5796193

[evad053-B64] Tomonaga S , Hirokane T, Awaya K. 1973. Lymphoid cells in the hagfish. Zoological Magazine. 82:133–135.

[evad053-B65] Traag VA , Waltman L, van Eck NJ. 2019. From Louvain to Leiden: guaranteeing well-connected communities. Sci Rep. 9:5233. 30914743 10.1038/s41598-019-41695-z PMC6435756

[evad053-B66] Weber JN , et al 2017a. Resist globally, infect locally: a transcontinental test of adaptation by stickleback and their tapeworm parasite. Am Nat. 189:43–57. 28035893 10.1086/689597

[evad053-B67] Weber JN , et al 2022. Evolutionary gain and loss of a pathological immune response to parasitism. Science 377:1206–1211. 36074841 10.1126/science.abo3411 PMC9869647

[evad053-B68] Weber JN , Steinel NC, Shim KC, Bolnick DI. 2017b. Recent evolution of extreme cestode growth suppression by a vertebrate host. Proc Natl Acad Sci U S A. 114:6575–6580. 28588142 10.1073/pnas.1620095114 PMC5488926

[evad053-B69] Witeska M . 2013. Erythrocytes in teleost fishes: a review. Zool Ecol. 23:275–281.

[evad053-B70] Wolf FA , Angerer P, Theis FJ. 2018. SCANPY: large-scale single-cell gene expression data analysis. Genome Biol. 19:15. 29409532 10.1186/s13059-017-1382-0 PMC5802054

[evad053-B71] Wynn TA . 2008. Cellular and molecular mechanisms of fibrosis. J Pathol. 214:199–210. 18161745 10.1002/path.2277 PMC2693329

[evad053-B72] Zheng GX , et al 2017. Massively parallel digital transcriptional profiling of single cells. Nat Commun. 8:14049. 28091601 10.1038/ncomms14049 PMC5241818

