# Supplementary material for: Single-Cell RNA Sequencing Reveals Microevolution of the Stickleback Immune System
Source: Genome Biol Evol. 2023 Apr 11;15(4):evad053. doi: 10.1093/gbe/evad053 (PMC10116603; doi:10.1093/gbe/evad053)
Supplement: evad053_Supplementary_Data [file evad053_supplementary_data.zip › SuppFigs.docx]

**SUPPLEMENTAL FIGURES**

**Single-cell RNA sequencing reveals micro-evolution of the stickleback immune system**

Lauren E. Fuess & Daniel I. Bolnick

**
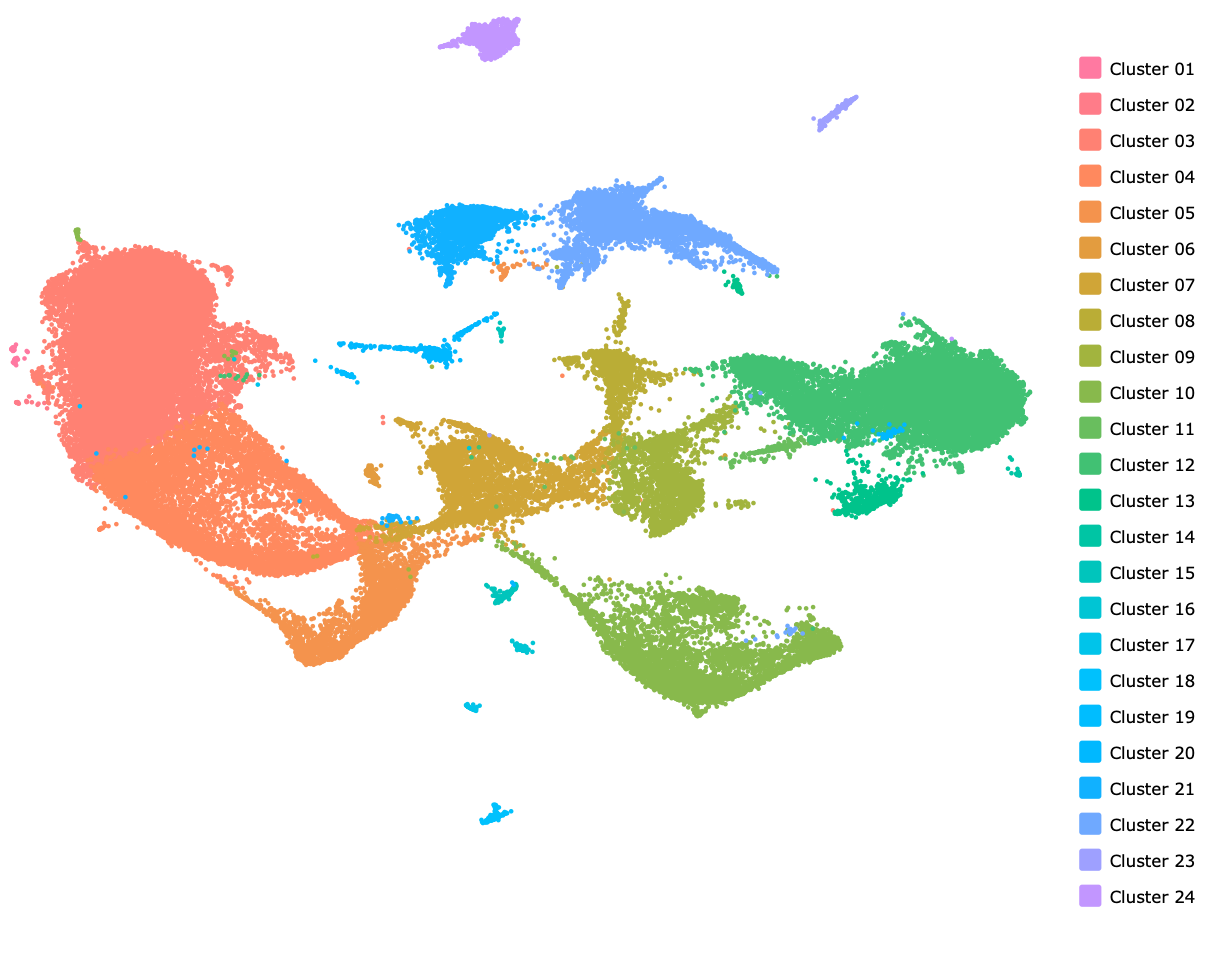
**

**Supplementary Figure 1:** Loupe uMap demonstrating original cluster assignments for each cell. Each individual dot represents the expression profile of a single sequenced cell

**Supplementary Figure 2:** Loupe uMap demonstrating cluster groupings condensed based on cell types. Five cell type clusters were comprised of multiple original clusters: neutrophils, RBCs, B-cells, HSCs, and NKCs. Grouped panels represent the spatial distribution of condensed clusters for each major cell type group. NKCs are shown in yellow with the cluster above HSCs and the cluster below HSCs representing independent original clusters.

**
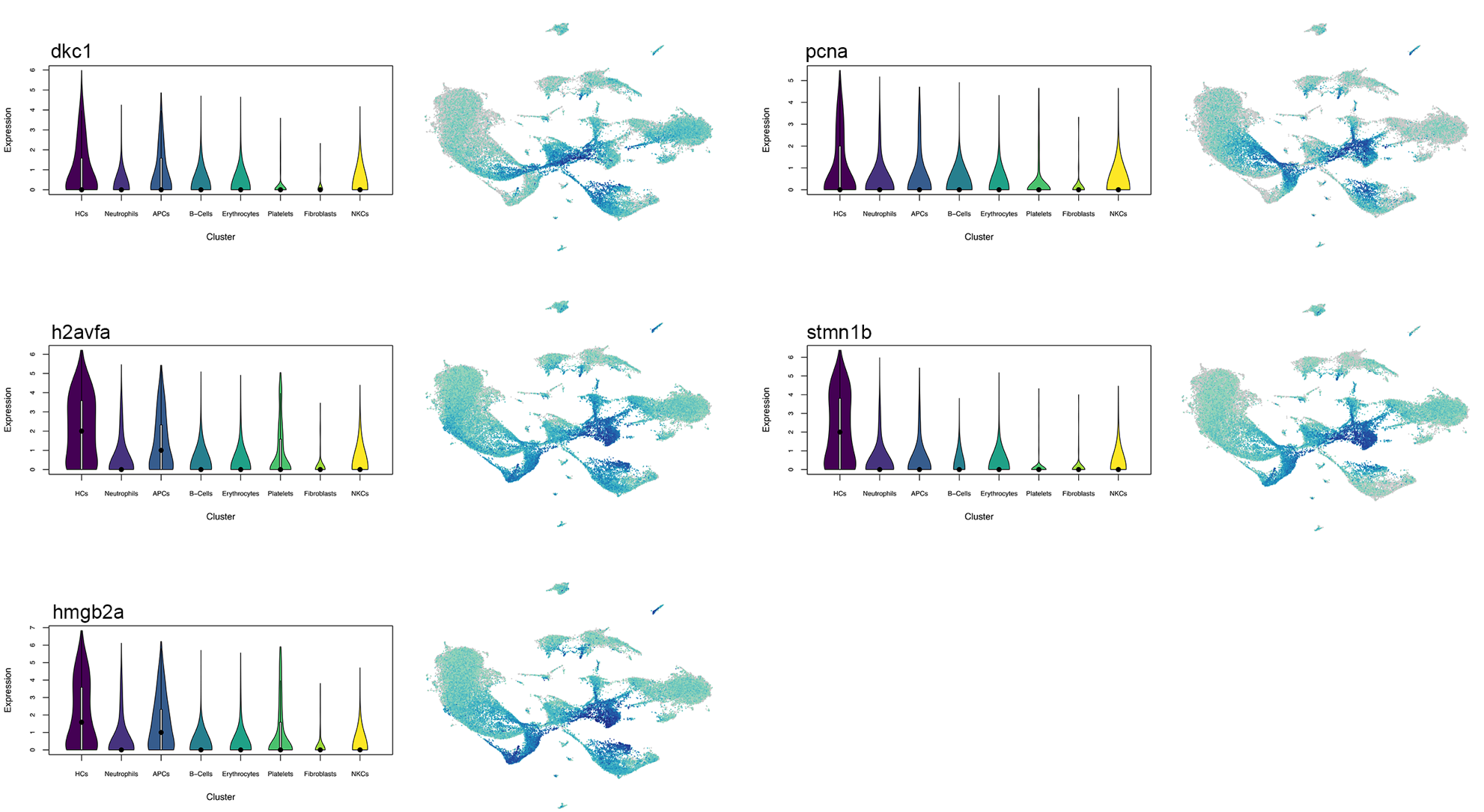
**

**Supplementary Figure 3:** Paired violin and Loupe uMap expression plots demonstrating patterns of expression for HC marker genes of interest. Plots display normalized (log-transformed) expression. Darker colors in cluster plots correspond to higher expression; each plot scaled independently

**
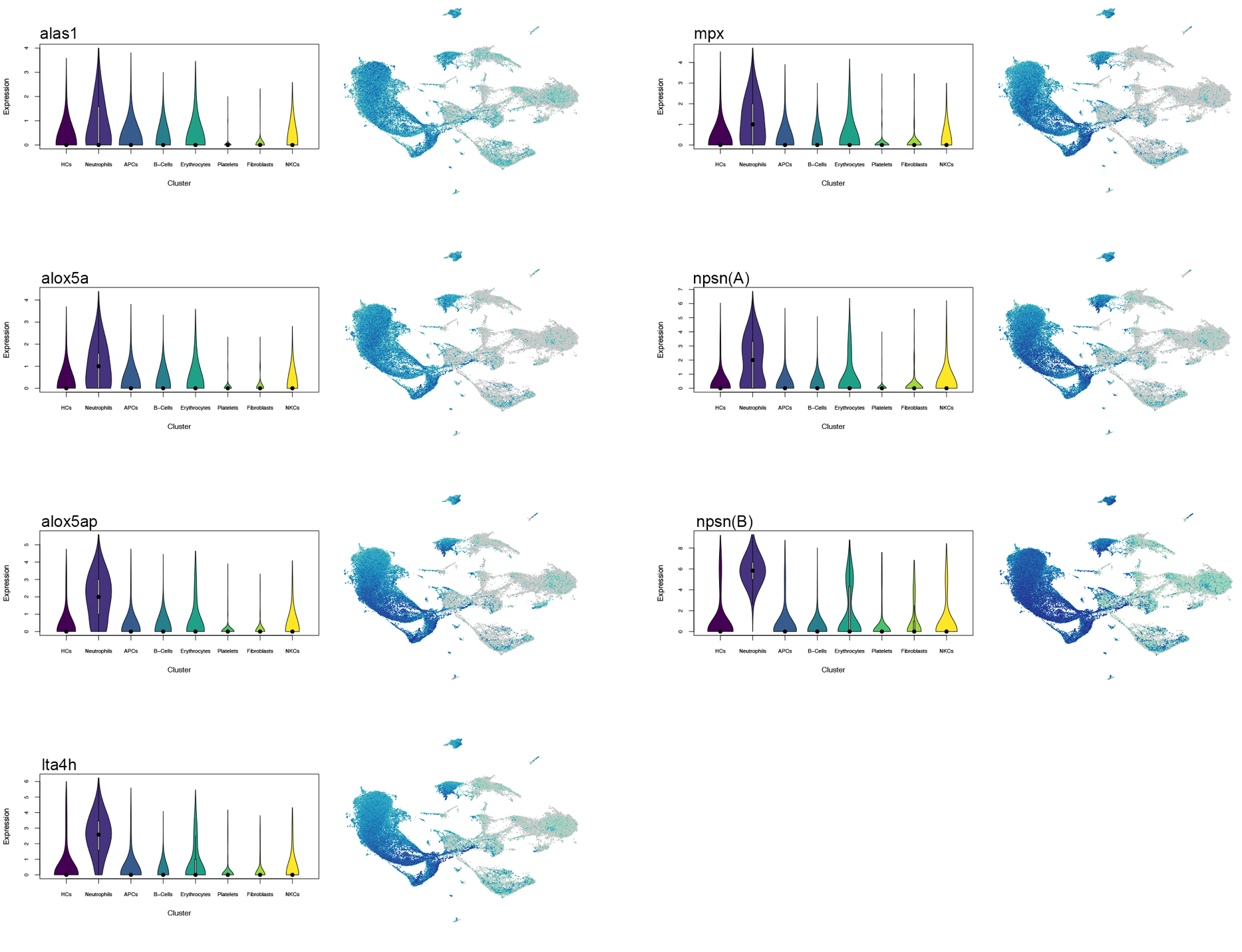
**

**Supplementary Figure 4:** Paired violin and Loupe uMap expression plots demonstrating patterns of expression for neutrophil marker genes of interest. Plots display normalized (log-transformed) expression. Darker colors in cluster plots correspond to higher expression; each plot scaled independently

**
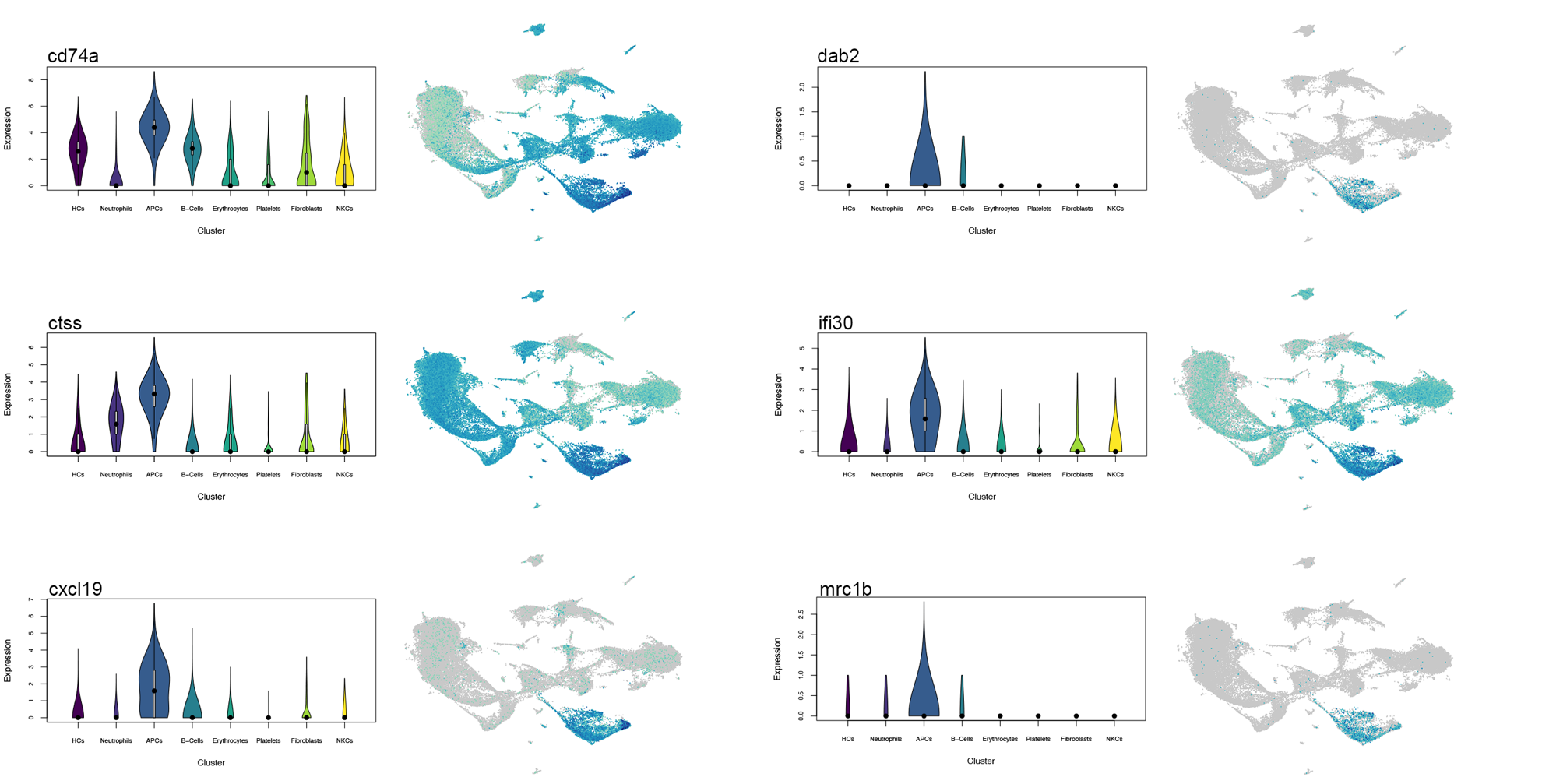
**

**Supplementary Figure 5:** Paired violin and Loupe uMap expression plots demonstrating patterns of expression for APC marker genes of interest. Plots display normalized (log-transformed) expression. Darker colors in cluster plots correspond to higher expression; each plot scaled independently

**
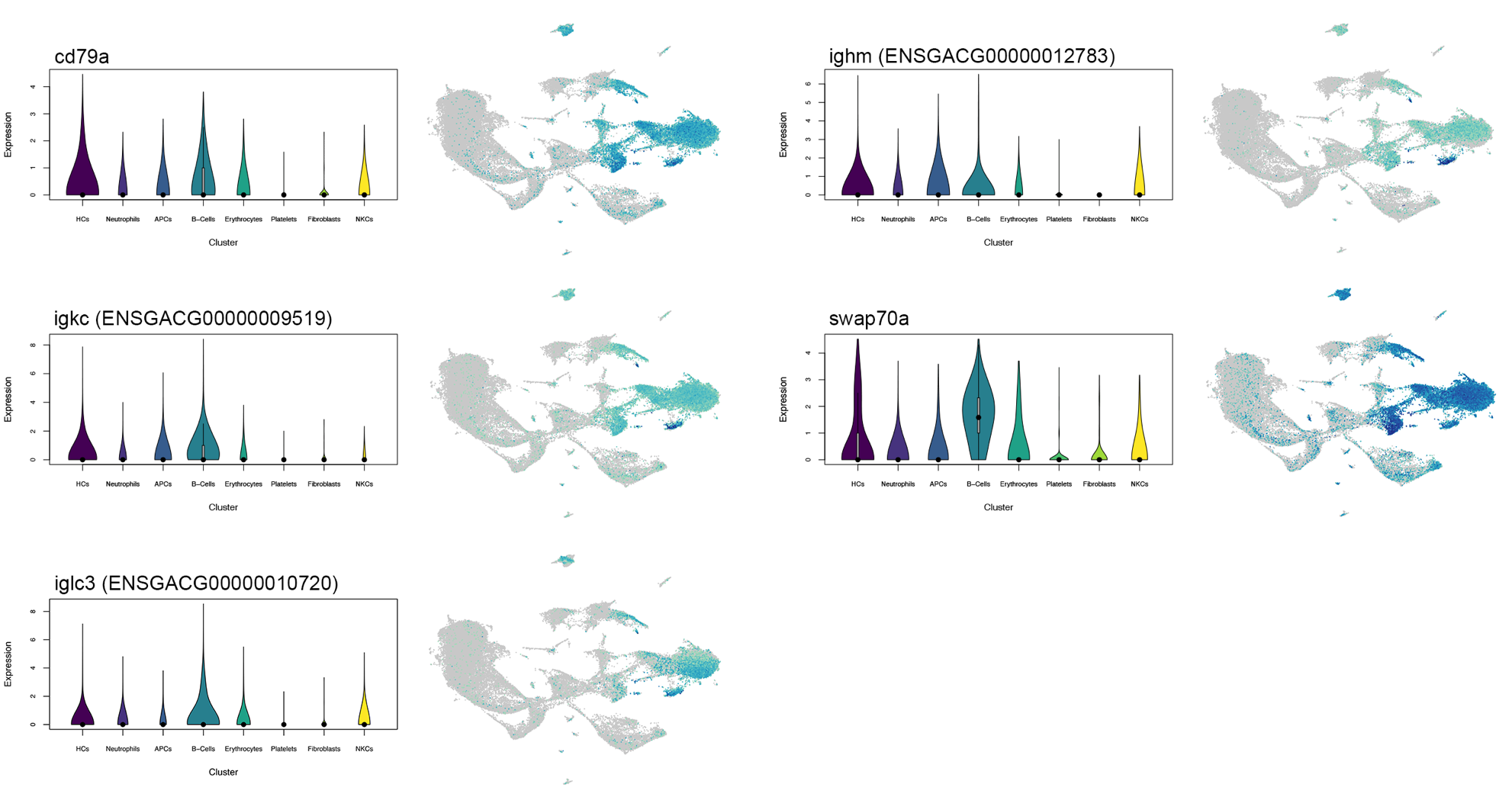
**

**Supplementary Figure 6:** Paired violin and Loupe uMap expression plots demonstrating patterns of expression for B-cells marker genes of interest. Plots display normalized (log-transformed) expression. Darker colors in cluster plots correspond to higher expression; each plot scaled independently

**
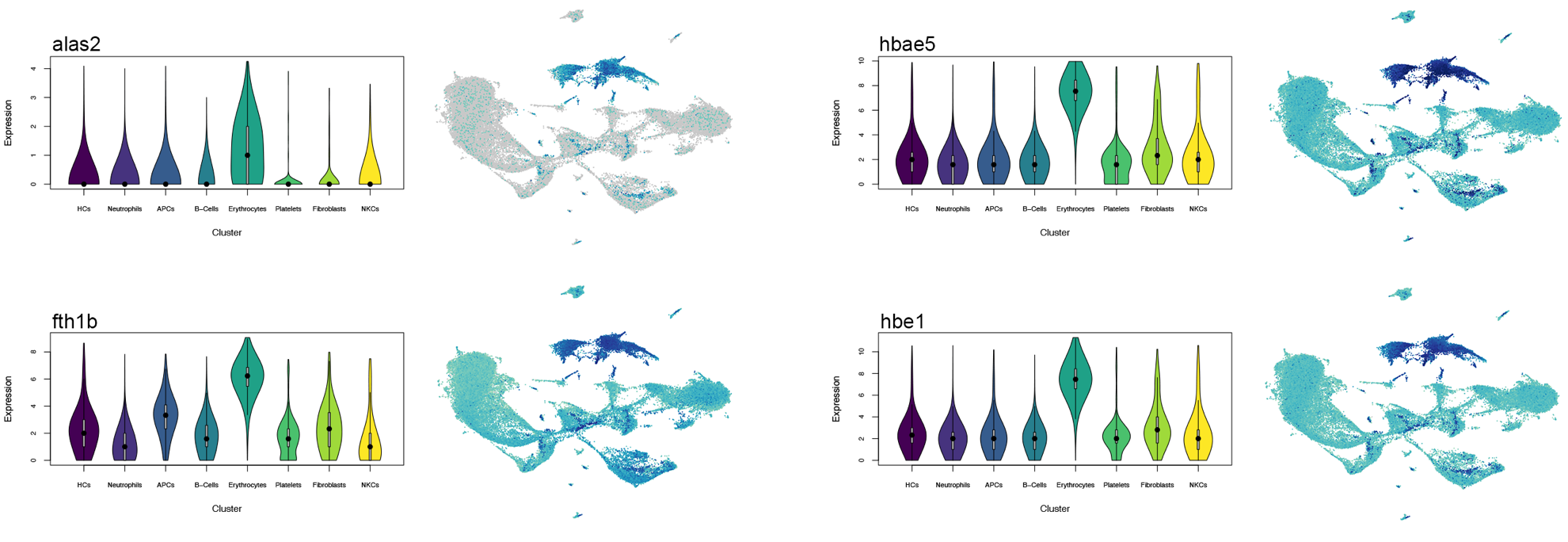
**

**Supplementary Figure 7:** Paired violin and Loupe uMap expression plots demonstrating patterns of expression for RBC marker genes of interest. Plots display normalized (log-transformed) expression. Darker colors in cluster plots correspond to higher expression; each plot scaled independently

**
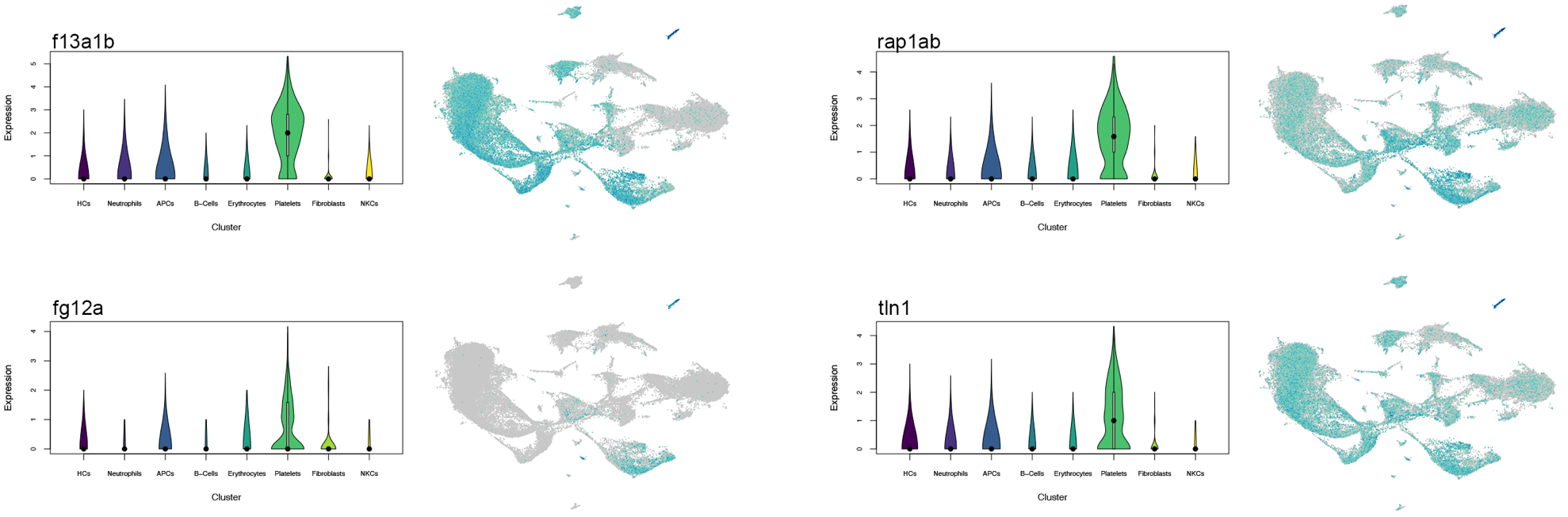
**

**Supplementary Figure 8:** Paired violin and Loupe uMap expression plots demonstrating patterns of expression for platelet marker genes of interest. Plots display normalized (log-transformed) expression. Darker colors in cluster plots correspond to higher expression; each plot scaled independently

**
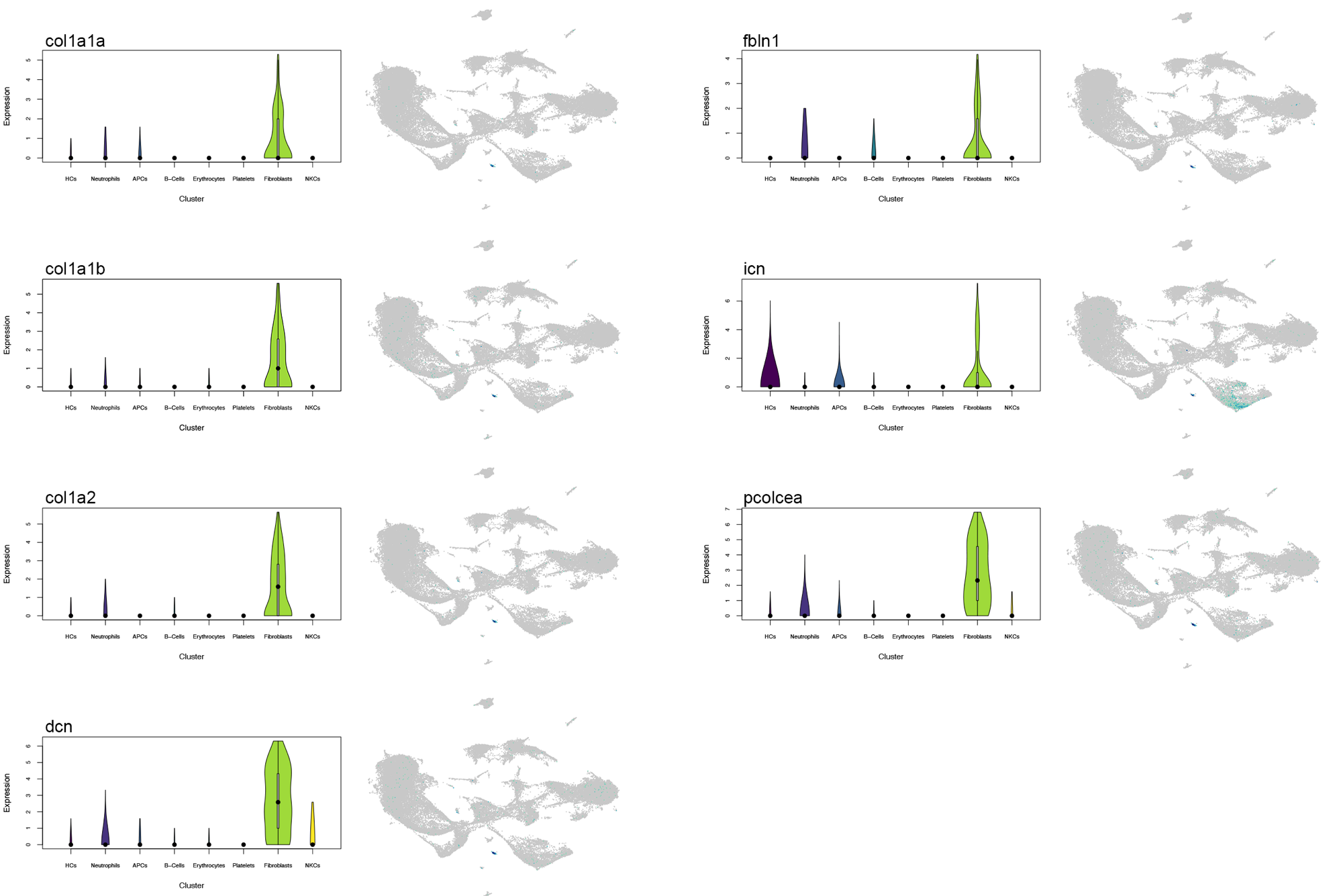
**

**Supplementary Figure 9:** Paired violin and Loupe uMap expression plots demonstrating patterns of expression for fibroblast marker genes of interest. Plots display normalized (log-transformed) expression. Darker colors in cluster plots correspond to higher expression; each plot scaled independently

**
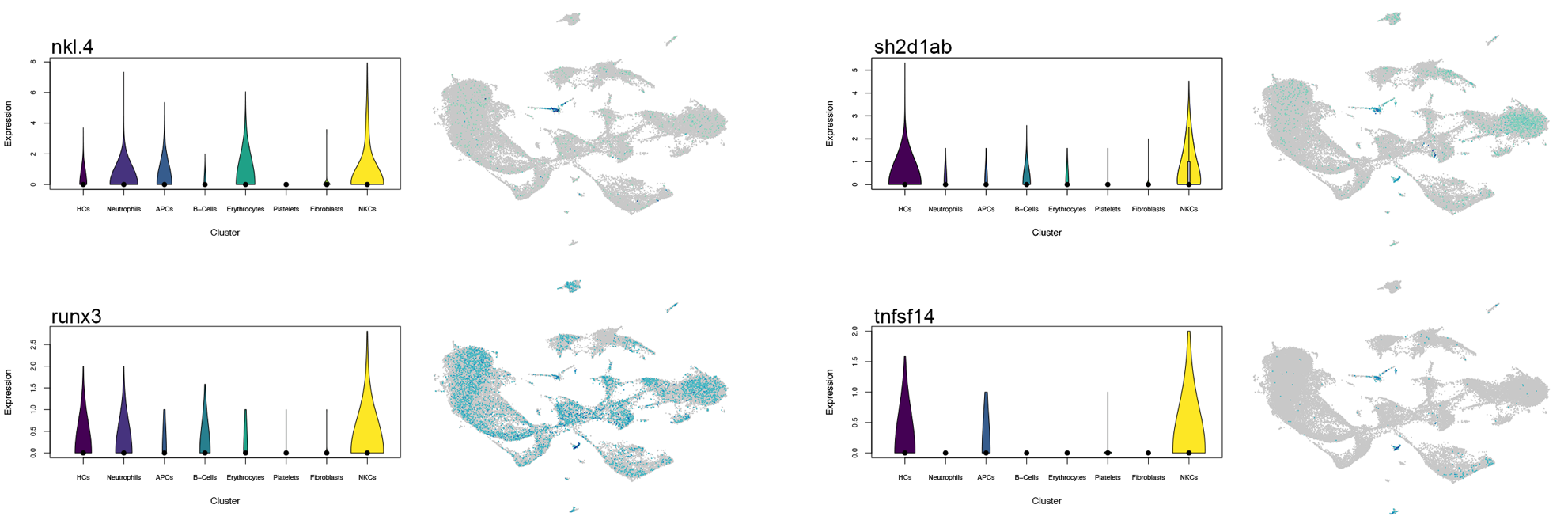
**

**Supplementary Figure 10:** Paired violin and Loupe uMap expression plots demonstrating patterns of expression for NKC marker genes of interest. Plots display normalized (log-transformed) expression. Darker colors in cluster plots correspond to higher expression; each plot scaled independently

**
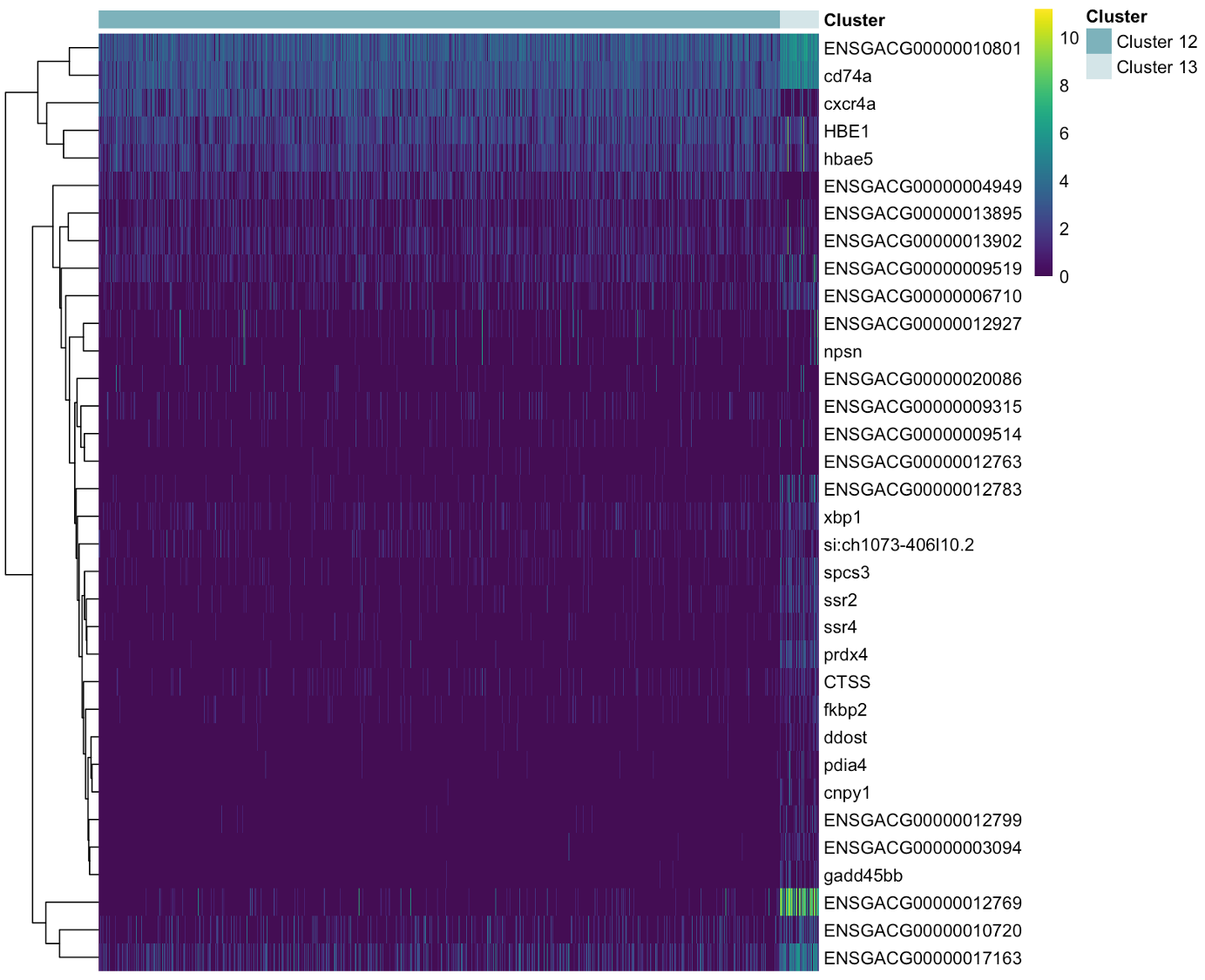
**

**Supplementary Figure 11:** Heatmap displaying normalized (log transformed) expression of genes which were significantly differentially expressed between the two major types of B-cells. Heatmap and gene dendrogram generated using the R package, pheatmap

**Supplementary Figure 12:** Alignment of predicted protein sequences of nephrosin from zebrafish, and the four predicted transcripts resulting from two nephrosin genes in the stickleback genome. Alignment generated using the R package msa.

**Supplementary Figure 13:** Pearson correlations between expression of identified lymphocyte or granulocyte markers and normalized lymphocyte or granulocyte frequency (detected by flow cytometry) in a previous transcriptomic study (Lohman et al. 2017). Regression line is shown in black and shading indicates 95% confidence intervals.

**Supplementary Figure 14:** Pearson correlations between expression of identified lymphocyte or granulocyte markers and normalized lymphocyte or granulocyte frequency (detected by flow cytometry) in a previous transcriptomic study (Fuess et al. 2020). Regression line is shown in black and shading indicates 95% confidence intervals.
